# Effects of Probiotic Supplementation during Chronic Rhinosinusitis on the Microbiome

**DOI:** 10.3390/jcm13061726

**Published:** 2024-03-17

**Authors:** Arkadiusz Standyło, Aleksandra Obuchowska, Anna Horaczyńska-Wojtaś, Grażyna Mielnik-Niedzielska

**Affiliations:** Chair and Department of Paediatric Otolaryngology, Phoniatrics and Audiology, Medical University of Lublin, 20-093 Lublin, Poland; a.standylo@gmail.com (A.S.);

**Keywords:** probiotic supplementation, microbiome, chronic rhinosinusitis, dysbiosis, paediatrics

## Abstract

Probiotics are live microorganisms that induce health benefits to the host. The consumption of probiotics can result in both prophylactic and therapeutic effects. Chronic rhinosinusitis (CRS) is an inflammatory condition that has a significant health and economic impact worldwide. Despite its great burden on the health-care system and patients’ quality of life, the variety of therapy options for CRS is currently limited. Inflammation, mucociliary dysfunction and changes in the microbial environment are thought to be the main factors causing the disease. Probiotics are a relatively new intervention, with a focus on the probiotic qualities and adaptive elements required for a bacterial strain to have a positive impact on CRS. The aim of this review was to review studies evaluating the potential beneficial effects of probiotics in the treatment of chronic rhinosinusitis. Future prospects and difficulties for probiotics in CRS are also highlighted.

## 1. Introduction

Chronic rhinosinusitis (CRS) is a significant healthcare burden and a major public health concern [1,2]. Chronic rhinosinusitis is the term used for rhinosinusitis that lasts for more than 12 weeks. It is characterised by two or more symptoms, one of which should be nasal obstruction or nasal discharge [2,3]. Children with predisposing conditions (such as allergic rhinitis, cystic fibrosis or anatomical structural abnormalities) are most likely to be diagnosed with this condition, which is associated with a significantly reduced quality of life [4]. The effects of local and systemic mechanisms and environmental factors can all contribute to the development of CRS in children. Both adults and children can be diagnosed with chronic rhinosinusitis, although in younger children the pathophysiology of the disease differs depending on the development of the immunological mechanisms involved in the inflammatory response [4].

CRS is characterised by inflammation of the paranasal sinus mucosa. CRS can be divided into CRS with nasal polyps (CRSwNP) and CRS without nasal polyps (CRSsNP), two main clinical phenotypes based on nasal endoscopy [2,5]. CRSwNP and CRSsNP are defined as rhinosinusitis lasting >12 weeks and are characterised by at least two of the following four symptoms; one of the highlighted main symptoms must be present: impaired nasal patency/blockage of the nasal cavities, catarrhoea (anterior/posterior), a painful feeling of craniofacial distension, impaired/loss of smell.

In addition, the diagnosis should take into account features of inflammation of the nasal mucosa, primarily in the middle nasal meatus, found during endoscopic examination of the nasal cavities and/or the presence of inflammatory lesions in the sinuses detected during CT of the paranasal sinuses. Any inflammatory process of the nasal mucosa without evidence of obvious nasal polyps should be treated as CRSsNP [2,5,6]. However, classifying CRS into CRSwNP and CRSsNP does not provide a complete understanding of the underlying pathophysiology [7].

CRS and CRS exacerbations (symptoms) are associated with a high burden as they require medical consultation, prescription of antipyretics, anti-inflammatory medication and/or antibiotics. In addition to direct costs, upper respiratory tract infections are associated with high indirect burdens due to parental absenteeism and a negative impact on the quality of life of children and their families [8]. 

Various treatment options exist for CRS, but there is a lack of broadly applicable and effective treatment approaches [1]. Saline nasal irrigations, systemic and/or topical corticosteroid therapy, antibiotic medication (typically macrolides because they also have anti-inflammatory properties), and functional endoscopic sinus surgery are the main treatment options now available [1,9,10,11]. Patients do not always react to available treatments though, and symptoms frequently return even after surgery.

According to recent research, the nasal and paranasal cavities of healthy people are colonised by commensal bacteria [10]. The mucous membranes of the sinuses are colonised by many commensal organisms, including fungi and both aerobic and anaerobic bacteria. The altered sinonasal microbiota, and sometimes also disruption of the nasal epithelial barrier, might be key factors in the pathology of CRS [1,3,12,13]. 

First- and second-hand smoking and environmental pollution can also affect the sinus microbiome by directly affecting the sinus epithelium, decreasing mucociliary clearance and increasing inflammatory markers, consequently affecting the diversity and bacterial richness of the microbiome, thus smoking affects the severity of CRS and may promote dysbiosis [10,14,15].

Pathogens or medications, such as frequent antibiotic courses, can disrupt this natural microbiota, resulting in an imbalance in the microbiome that favours the emergence of CRS [11,16,17]. Following surgery and frequent antibiotic administrations, the microbiome’s composition continues to alter, and antibiotic resistance increases [11,17]. This results in uncontrolled repopulation of empty niches, which may interfere with the restoration of the “optimal” microbial community [17].

The frequent use of antibiotics to treat childhood respiratory infections, which kill saprophytic bacteria living on the mucous membranes of the upper airways, is a factor that favours the development of bacterial biofilm in the nasopharyngeal space. In addition, inflammation is maintained to a large extent by the bacterial biofilm that forms, according to recent research [18]. Biofilm is found in the nasopharynx of paediatric patients with chronic diseases of the upper respiratory tract [19]. Diagnostics in the paediatric population are associated with the need to take into account numerous factors that are important in the development of CRS.

## 2. Methods

**Figure 1:**
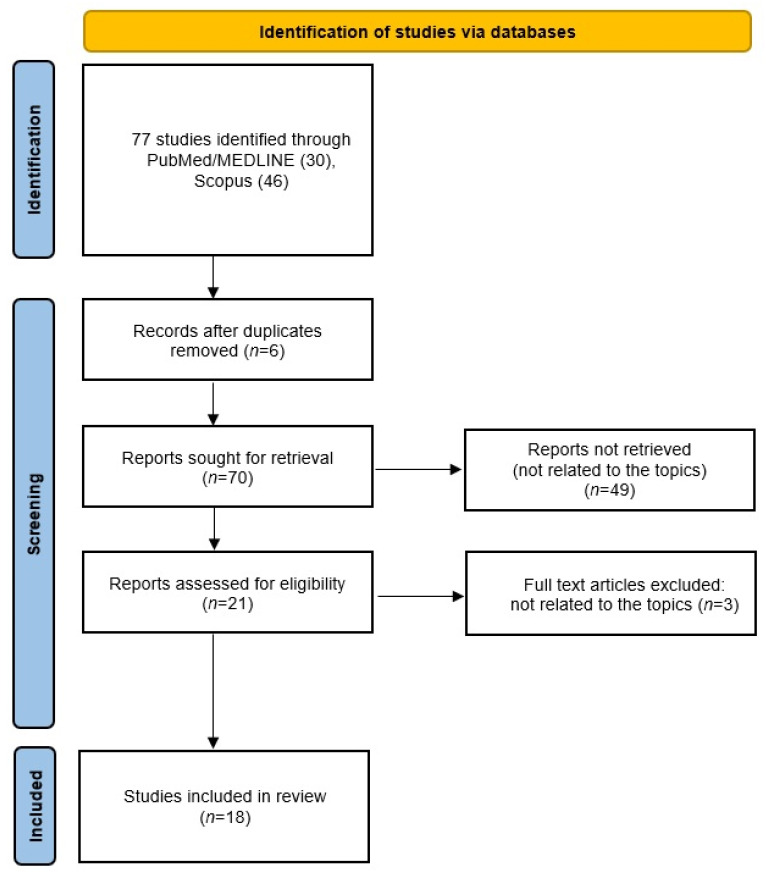
Flow diagram of the literature search. The Preferred Reporting Items for Systematic Reviews and Meta-Analyses (PRISMA) flow diagram shows the study selection process.

This study was conducted according to the Preferred Reporting Items for Systematic Reviews and Meta-Analyses (PRISMA) statement published in 2020. A flow chart is shown in Figure 1. The literature search for this review was conducted using the following keywords: “probiotic supplementation”; “microbiome”; “chronic rhinosinusitis”; “dysbiosis”; “paediatric”. Two electronic databases were searched: MEDLINE (via PubMed) and Scopus. The last search in each database was performed on 21 December 2023. The authors also conducted a ‘snowball’ search to identify additional studies by searching the reference lists of publications eligible for full-text review; however, no additional records meeting the inclusion criteria were identified. It was not necessary to contact the authors of the retrieved research articles for additional information. Duplicates were removed using Mendeley’s automated duplicate search engine, followed by a manual search. The results of relevant studies published in English are summarised and discussed in this coherent review.

## 3. Probiotics in Chronic Rhinosinusitis

The definition of probiotics by the International Scientific Association for Probiotics and Prebiotics (ISAPP) panel of experts defines them as “live microorganisms which, when administered in adequate amounts, confer a health benefit on the host” [16,20].

Probiotics, prebiotics, and related microbiome studies have made significant strides in recent years [21,22,23,24]. Probiotics and prebiotics are tools for managing the microbiota and enhancing host health and have attracted more attention recently as tools for manipulating the microbiome, along with the popularity of microbiome research [25]. Probiotic strains function in a number of ways, such as modifying immune response, generating organic acids and antimicrobial substances, and interacting with the host and resident microflora [25,26]. Probiotics’ capacity to stop the overgrowth of pathogens can thus be viewed as having a significant positive effect [7,27]. 

The therapeutic potential of probiotics has aroused curiosity as an advanced oral and/or nasal treatment option for long-term respiratory conditions. Probiotics have been shown to have antiviral effects. This is true for common respiratory viruses such as influenza, rhinoviruses, respiratory syncytial virus and coronaviruses [28,29,30]. The relevant probiotic modes of action can be broken down into at least three categories: control of microbial interactions, such as such as colonisation, toxin production, inflammation, and biofilm formation; immunomodulation, and epithelial barrier protection [1,31]. The effects of probiotics on immunomodulation, barrier protection, and antipathogenic action vary depending on where in the body they are found. [1]. 

Potential probiotic bacteria should be distinguished by specific adaptation mechanisms. First, a strong ability to adhere to the nasal epithelium to cope with mucociliary clearance as well as to compete with pathobionts such as *S. aureus* should be established. An important aspect is also the specific environmental conditions in the upper respiratory tract (URT), characterised by higher oxygen concentrations (partial pressures of up to 160 mmHg pO_2_), a lower temperature and a nasal cavity pH of about 6.3, whereas the nasopharynx has a slightly higher pH (pH 7) [1,27,32].

Inhibiting pathogen growth is frequently considered a key probiotic benefit. Producing antimicrobial compounds or engaging in competitive exclusion—a strategy for overcoming resource and binding site constraints—can accomplish this [1,27]. Probiotic microbe-associated molecular patterns (MAMPs) and pattern recognition receptors (PRRs) of host epithelial and immune cells can interact directly to produce immunomodulatory effects, or microbial soluble factors and metabolites can be released into the environment to activate signalling cascades in host cells. Probiotics differ in their effect on immune cells and/or epithelial cells because the total amount of MAMPs expressed by a particular strain varies from strain to strain. PRRs on epithelial and innate immune cells detect MAMPs [33]. Some of the most studied PRR receptors are Toll-like receptors (TLR) [33]. Alterations in TLR expression have been reported in patients with CRS, thus dysregulation of TLR2 function can lead to chronic inflammation [34]. However, the interactions between TLR receptors and lactic acid bacteria in the upper respiratory tract are still poorly understood [35,36]. 

Some probiotic strains may improve and/or control epithelial barrier function, and it is worth mentioning that probiotics can interact with epithelial cells’ PRRs [1,33,37]. Disrupting the nasal epithelial barrier leads to infiltration of the submucosal space, which can lead to an immune cascade in the nasal mucosa. As a physical barrier, the nasal epithelium separates the internal and external environments and is important for protection against allergens, pathogens and other irritants. It refers to tight junctions (TJ), adherent junctions (AJ), desmosomes and others. Studies show impairment of the TJ and AJ proteins in CRS [38]. The microbiota of the nasal mucosa influences protection against external stimuli and modulates immunity [38].

*L. casei AMBR2* can enhance epithelial barrier immunity by modulating the microbiota [39]. In a study by Martens et al., the ability of *L. casei AMBR2* to restore epithelial barrier function was observed in vitro, mainly through the re-organisation of the tight-junction (TJ) proteins [34]. In one study, *L. casei AMBR2* was shown to both increase transepithelial electrical resistance (TEER) and block IL-4-induced nasal mucosal permeability. In addition, *L. casei AMBR2* promoted the recombination of the tight junction (TJ) proteins occludin (OCLN) and zonula occludens (ZO-1), which play a crucial role in binding and signalling transduction [34]. Moreover, lactic acid bacteria interact with receptors, such as TLRs, that are present on the epithelium [34,39]. When TLRs are activated, they can trigger signalling cascades that mount an immunological defence against identified pathogens [39]. Lactic acid bacteria promote nasal epithelial barrier function through reorganisation of TJ expression, which is dependent on TLR2-TLR6 signalling [34]. This type of interaction can restore the damaged epithelium barrier by modulating AJ and TJ.

Further studies are needed to demonstrate an interaction between probiotics and PRRs using the sinus epithelium, as well as a study of the modulating effect of probiotics on Toll-like receptors (TLRs) and the corresponding cytokines in chronic rhinosinusitis.

## 4. Microbiome in Chronic Rhinosinusitis

There is evidence that the complex relationship between the human host and the microbiota is a determinant of human health. The microbiome is made up of a variety of organisms that are known to benefit the development and health of the host [40,41,42]. Research has shown that the gut microbiota is crucial for maintaining the health of the host [23,43,44]. However, the microbiota of the upper airways has received little research attention [45,46]. The absence of beneficial bacteria and the presence of pathogenic microorganisms are two characteristics of long-term inflammatory diseases of the nose and sinuses, such as allergies and chronic sinusitis, which have only recently come to the attention of researchers [46,47].

Dysbiosis is an imbalance in our microbiota’s composition and metabolic activity which can be defined as a loss or increase in the number of bacteria that are health-promoting or disease-promoting, respectively [40,48]. It is characterised by the loss of beneficial commensal bacteria that help prevent opportunistic pathogens from overgrowing [1,26,49]. Different parts of the body can experience functional or compositional perturbations in the microbiota, and this dysbiosis has been linked to a number of diseases, i.e., CRS, asthma, Crohn’s disease, and ulcerative colitis [40,48,49,50,51]. Different bacterial strains interact differently with different types of host cells and mucus, with different elements of the innate and adaptive immune systems of the respiratory system, with exposure to medical treatments, and with rival microbial species [1,40]. It is important to identify factors that may influence the composition, stability and resilience of the sinus microbiota [10]. The risk of developing chronic diseases may be increased by the loss of microbiome diversity, interestingly, and it is apparently associated with damage to the epithelial barrier. Dysbiosis of the microbiome is thought to be a key biomarker of chronic rhinosinusitis and trigger of disease progression [38,50,52].

The microbiome of CRSsNP differs significantly from healthy controls, whereas the microbiome of CRSwNP does not show clear differentiation [53]. In study by Boeck et al., it was observed that patients with CRSsNP had lower bacterial diversity than patients with CRSwNP [7]. In other published studies, *Staphylococcus, Alloiococcus* and *Corynebacterium* were more common in patients with nasal polyps, whereas *Streptococcus, Haemophilus* and *Fusobacterium* were more common in those without nasal polyps [54,55]. It is likely that changes in microbiome diversity play a greater role in the disease of CRSsNP patients than CRSwNP patients [7,53]. Alterations in the bacterial community might be a crucial factor in the development of CRS [53].

Pathogenicity is strain-specific; thus, distinct strains may differ in virulence components, which can lead to varying levels of pathogenicity [31]. The normal upper respiratory tract microbiota of healthy adults and/or children has been described to include *Lactaseibacillus, Dolosigranulum*, and *Lactococcus* species [1,56,57]. However, a 2-year-old child’s respiratory microbiome is different from an adult’s in terms of composition and diversity [58]. *Lactobacilli* are detected in nasopharynx and tonsillar crypts of children and adults [1,27,59].

Different types of *Lactobacillaceae*, such as *Lactiplantibacillus*, *Latilactobacillus*, and *Lactaseibacillus*, were discovered to be more prevalent and abundant in healthy patients compared to CRS patients in a microbiome comparison study between healthy controls and CRS patients conducted by De Boeck et al. [1,27]. *Staphylococcus*, *Corynebacterium*, *Propionibacterium*, *Dolosigranulum*, and *Streptococcus* species are frequently identified in the upper respiratory tract of healthy people who do not exhibit any overt symptoms [32,50,60,61]. Pathogenic bacteria such *Moraxella catarrhalis*, *Haemophilus influenzae*, *Streptococcus pneumoniae*, and *Staphylococcus aureus* seem to be more common or frequent in upper respiratory tract disorders [1,10,16,31,45,60,61]. *Staphylococcus aureus*, *Haemophilus influenzae* and *Moraxella catarrhalis* are the main pathogens of CRS and are able to occupy a dominant niche as pathobionts; thus, these profiles are at risk of developing both acute and chronic illness [10,61].

Early life observations of children suggest that the combination of *Dolosigranulum*, *Moraxella* and *Corynebacterium* forms a more stable microbiome than profiles dominated by *Streptococcus* and *Haemophilus* [50,60,61]. However, children’s nasal microbiomes are more dense but less diverse compared to adults. Stable microbiome composition and respiratory health were associated with microbiome profiles characterised by *Corynebacterium* and *Dolosigranulum* species in early life and *Moraxella* species at 4–6 months of age [32,58,62]. However, frequent antibiotic use was characterised by a reduced abundance of presumably beneficial commensal bacteria such as *Dolosigranulum* spp. and *Corynebacterium* spp. in the upper respiratory tract of healthy children [32].

One study showed that samples from CRS patients were functionally less diverse than samples from healthy patients and significantly enriched for bacterial virulence pathways and antimicrobial metabolite production [63]. The likelihood of developing acute otitis media appears to be inversely associated with *Moraxella* in children who had higher abundances of taxa like *Lactococcus* and *Dolosigranulum* [58,64].

Interestingly, in study by Biesbroek et al., they observed that early colonisation of *Moraxella* and *Dolosigranulum* in association with *Corynebacterium* was a feature of more stable respiratory microbiome profiles in the first two years of life, whereas microbiome instability was associated with microbiome profiles being dominated by *Haemophilus* and *Streptococcus*. A more stable microbiome composition over time was produced by early colonisation with *Moraxella* or *Dolosigranulum*/*Corynebacterium* dominance. [58]. It was discovered that breastfeeding affected *Dolosigranulum* and *Corynebacterium* colonisation. This was connected to a significant decrease in upper respiratory tract infections and the microbiome profile. Given that *Dolosigranulum* is a lactic acid bacterium and that breastfeeding has protective benefits, it has been demonstrated that this microbiome profile may be advantageous to respiratory health [58].

The quantity of lactobacilli in URT is significantly less than other locations in the human body [27,56], but this does not exclude their potentially health-promoting role. The more established strains, notably those that belong to the *Lactobacillaceae* family, are already widely utilised and have a known safety profile. It has been proposed that lactic acid bacteria (LAB) can rebuild commensal microbiomes [16,27,65]. It is worth noting that LAB are part of the microbiome of the upper respiratory tract [31,60,62]. Bacterial taxa with potential beneficial effects on the upper respiratory microbiota and noticeable probiotic potential include *Streptococcus* or *Dolosigranulum* taxa [33]. Though members of the Dolosigranulum genus are relatively unknown LAB, *Dolosigranulum* and *Corynebacterium* have recently attracted more attention due to their apparent dominance within the nasopharyngeal microbiota [31,60,61,66].

## 5. Therapeutic Applications of Lactic Acid Bacteria Probiotics

Probiotics are mainly used to improve gastrointestinal health, and there have been many promising clinical results so far [25]. LAB have been researched as the majority of probiotics thus far (order *Lactobacillales*) [1,67]. Since more than 100 years ago, *Lactobacilli* have been used safely in dairy products, fermented meals, and nutritional supplements on a regular basis. As a result, they make an intriguing probiotic choice.

There is currently inadequate understanding of sinonasal dysbiosis and its significance in the pathophysiology of the disease to allow for the development of any therapeutic that could restore healthy microflora [17]. A review study by Man et al. showed that a balanced airway microbiota plays an important gatekeeping role in respiratory health [32]. Clinical studies investigating the potential of topical probiotics are limited. It appears that there are many important concerns that remain unsolved about the true function of bacteria in CRS. LAB potential for use as probiotics in CRS is still largely unexplored [1,67,68]. The research suggests that lactic acid bacteria are deficient in CRS.

In a double-blind, randomised, parallel-group, placebo-controlled study conducted by Garaiova et al., a probiotic-based supplement was given daily to a group of adolescents for six months [44]. The children were given one chewable tablet a day containing Lab4 probiotic consortium: *Lactobacillus acidophilus CUL21* (NCIMB 30156) and *CUL60* (NCIMB 30157), *Bifidobacterium bifidum CUL20* (NCIMB 30153) and *Bifidobacterium animalis* subsp. *lactis* *CUL34* (NCIMB 30172). With daily use of a probiotic-based supplement, the authors found a significant reduction in cough frequency, absenteeism and antibiotic use [44]. According to a study by King et al., newborns and children treated with probiotics had a 29% lower relative risk of needing antibiotics to prevent acute respiratory and gastrointestinal infections [69].

A study by Andaoro et al. showed that children had fewer and shorter episodes of GABHS pharyngitis and tonsillitis after a 90-day course of oral probiotic spray containing *S. salivarius* and *S. oralis* [8]. The administration of a probiotic mixture containing *Lactobacillus plantarum LP01*, *Lactobacillus lactis* subspecies *cremoris LLC02* and *Lactobacillus delbrueckii* subspecies *delbrueckii* significantly reduced the intensity of symptoms in patients with acute and chronic pharyngotonsillitis already receiving antibiotic treatment, according to clinical experience reported in a study by Albanese et al. [70]. Two other studies found that the same LAB-containing preparation used in the previous study reduced symptoms in patients with acute and chronic otitis media and laryngotracheitis [71,72].

Interestingly, in a study by Rosas-Salazar et al. investigating the role of the nasopharyngeal microbiome in the development of wheezing in children, it was noted that increased *Lactobacillus* abundance in infancy was associated with a reduced risk of wheezing in children at 2 years of age [62]. In their study, the authors suggest that the provision of *Lactobacillus* bacteria during acute respiratory infections in infancy may have an impact on the prevention of future disease development [62].

Because of its significant abundances, which can reach up to 50% in the healthy upper respiratory tract in children, the understudied species *Dolosigranulum pigrum* has particularly drawn attention as a possible next-generation probiotic [27,58,73]. In a study by Gan et al. and De Boeck et al., the middle meatus of the control group had a considerably higher abundance of the species *Dolosigranulum* than those of CRS patients [7,73]. *Dolosigranulum* was also shown to be more prevalent in the nasopharyngeal microbiomes of healthy children than in those who had recurrent acute otitis media [74].

In study by Mårtensson et al., the impact of LAB spray on CRS patients who did not have nasal polyps was examined. They administered a honeybee nasal spray containing LAB species (nine *Lactobacillus* and 4 *Bifidobacterium* species) microbiota intranasally to patients with CRS for two weeks. They demonstrated that the probiotic spray was well-tolerated and safe to administer, but they did not see a favourable effect of the spray on the course of the disease. There was no difference in the nasal microbiome between LAB and sham, neither for commensals nor CRS pathogens after treatment with LAB and sham [16]. However, they reported antimicrobial activity against pathogens such as *Streptococcus pyogenes*, *Staphylococcus aureus*, and *Pseudomonas aeruginosa* [16]. This research marks a significant advance in the use of nasal probiotic sprays for the treatment of CRS.

According to a study by van den Broek, *L. rhamnosus GG* can stop *M. catarrhalis* from growing, showing antimicrobial activities [64]. These antimicrobial activities against *M. catarrhalis*, *H. influenzae*, and *S. aureus* are similarly seen for *L. casei AMBR2*, as well as for strains of *D. pigrum* against *S. aureus* [27,75].

De Boeck et al. demonstrated that in the primary cells of CRS patients, *the L. casei* isolate *AMBR2* could repair the breakdown of the airway epithelial barrier [27]. In another work by De Boeck et al., the *L. casei AMBR2* strain in a nasal spray was tested on healthy participants, and the results showed that the strain was safe and capable of temporarily colonising the URT. They demonstrated that *L. casei AMBR2*, which was isolated from the URT, had superior qualities in terms of oxidative-stress endurance and fimbriae structures. It was also demonstrated that *L. casei AMBR2* was well-tolerated and could adapt to and colonise the human nasopharynx [1,27]. Moreover, the *L. casei AMBR2* strain from the nasopharynx is catalase-positive, whereas most other *Lactobacillus* species are catalase-negative, suggesting a role for catalase in adaptation to the upper respiratory tract environment [31]. *L. casei AMBR2* is a promising candidate for a live biotherapeutic product as a probiotic. It has the ability to inhibit the growth and inflammatory properties of pathogens such as *Staphylococcus aureus* and to promote epithelial barrier function [34,65]. Thus, there is a lot of interest in further using this strain in CRS patients.

Only a small number of studies examined the effects of topical probiotic therapy in CRS patients, using probiotic sinus irrigations [12] or probiotic nasal sprays [16]. Since 2018, the probiotic preparation *Lactococcus lactis W136*, which is marketed as a probiotic, was made available in sachets for topical nasal and sinus rinses in the US and Canada [42]. In these studies, no difference was noticed between the test group and the control group, but a possible explanation may be that the LAB strains used are not adapted to URT [16]. Interestingly, the study discovered that nasal irrigations with *L. lactis W136* were well-tolerated in patients with CRS. During and up to two weeks after treatment, significant improvements in symptoms and quality of life were seen across a number of parameters, including the Sino-Nasal Outcome Test 22 (SNOT-22) [12]. In a pilot study by Endam et al. [12], intranasal irrigation of live *Lactococcus lactis W1366* was performed in patients with refractory chronic rhinosinusitis. The study demonstrated the topical safety of *L. lactis W136* in patients with CRS. The study reported clinical benefits in terms of symptom reduction in patients; however, few significant changes in the microbiome profiles of CRS patients were observed following treatment. Therefore, both studies highlight the potential for local LAB administration in CRS, but they also point out that careful consideration must be given to probiotic strain selection.

Based on the few sample size studies completed thus far, EPOS2020 (European Position Paper on Rhinosinusitis and Nasal Polyps) recently came to the conclusion that there is presently no evidence to support the use of oral or topical URT probiotics as a therapy option for people with CRS [2]. Noting the specificity of probiotic strains and multifactorial mechanisms of action, which also depend on the site of administration, it should be noted that the analysis of the use of probiotic preparations is a significant challenge and generalising their effectiveness based on the sparse number of studies available is difficult.

In general, the safety profile of next-generation probiotics, such as the more recently proposed *D. pigrum*, is less well-known, and additional clinical investigations are required. It is proposed that the taxa *Lactobacillus* and *Dolosigranulum* should be used as health indicator taxa [1]. Because different strains and species have distinct virulence properties, the link between health and disease should be investigated at the strain or species level [31].

## 6. Conclusions

The upper tract microbiome may be a previously unrecognised and potentially modifiable mechanism by which it influences susceptibility and respiratory symptoms. Research into microbiome dysfunction and its impact on the development of CRS may clarify the causal relationship between microbiome imbalance and the host inflammatory response.

LAB merit further research in the area of microbial barrier modulation for CRS. The Lactobacillaceae and Dolosigranulum taxa hold promise for further investigation as potential probiotics for the treatment of CRS. Targeting the microbiome with probiotic LAB bacteria will receive more attention in the future as awareness of the negative effects of widespread antibiotic use increases. It is important to note that probiotics are not expected to be a cure for CRS, but rather to relieve symptoms or prevent exacerbations of the disease.

The above studies highlight the difficulty in identifying potential probiotic microorganisms for therapeutic use. There is a need for large, well-controlled clinical trials to demonstrate the impact of probiotic use on changes in the microbiome of CRS patients and potential changes in clinical symptoms and inflammatory outcomes.
